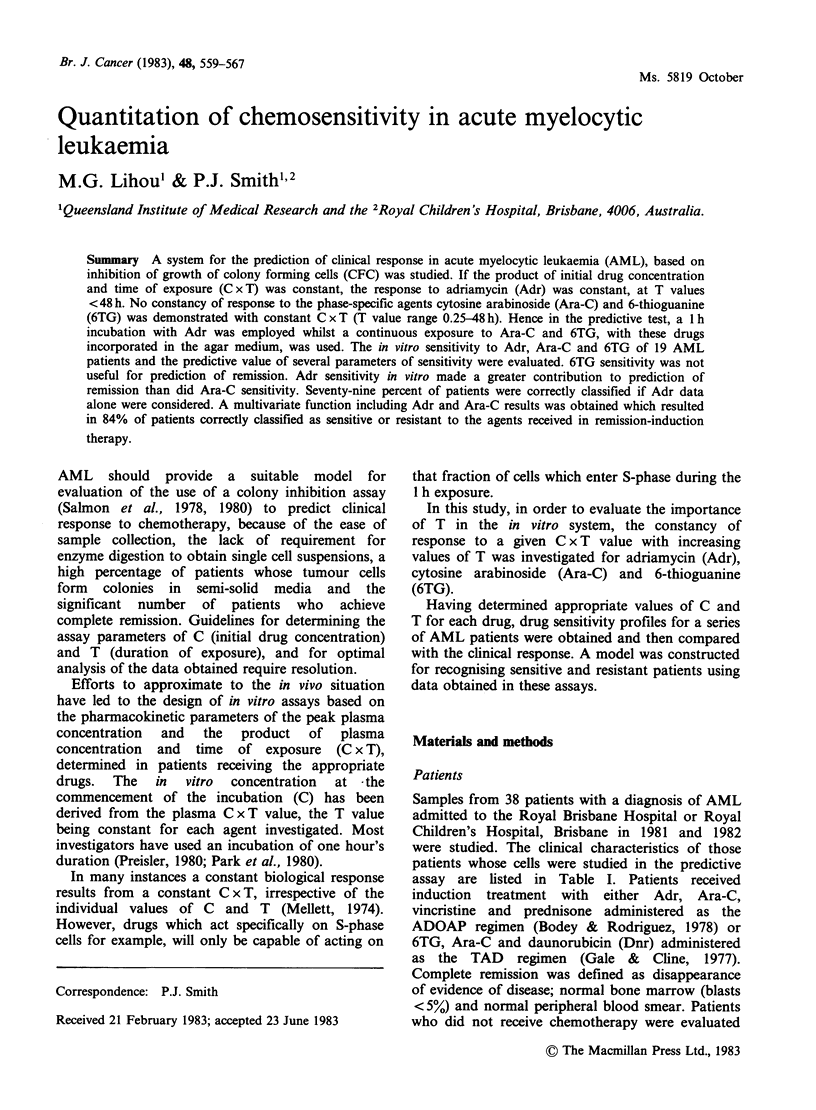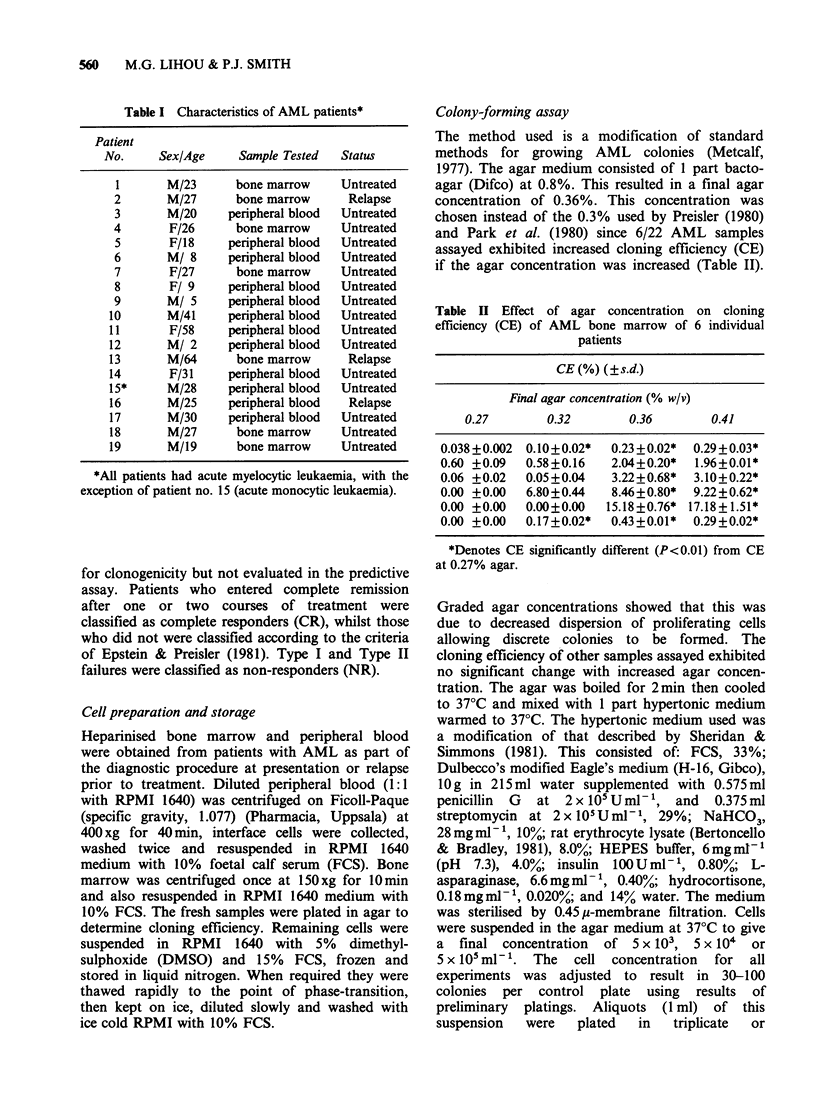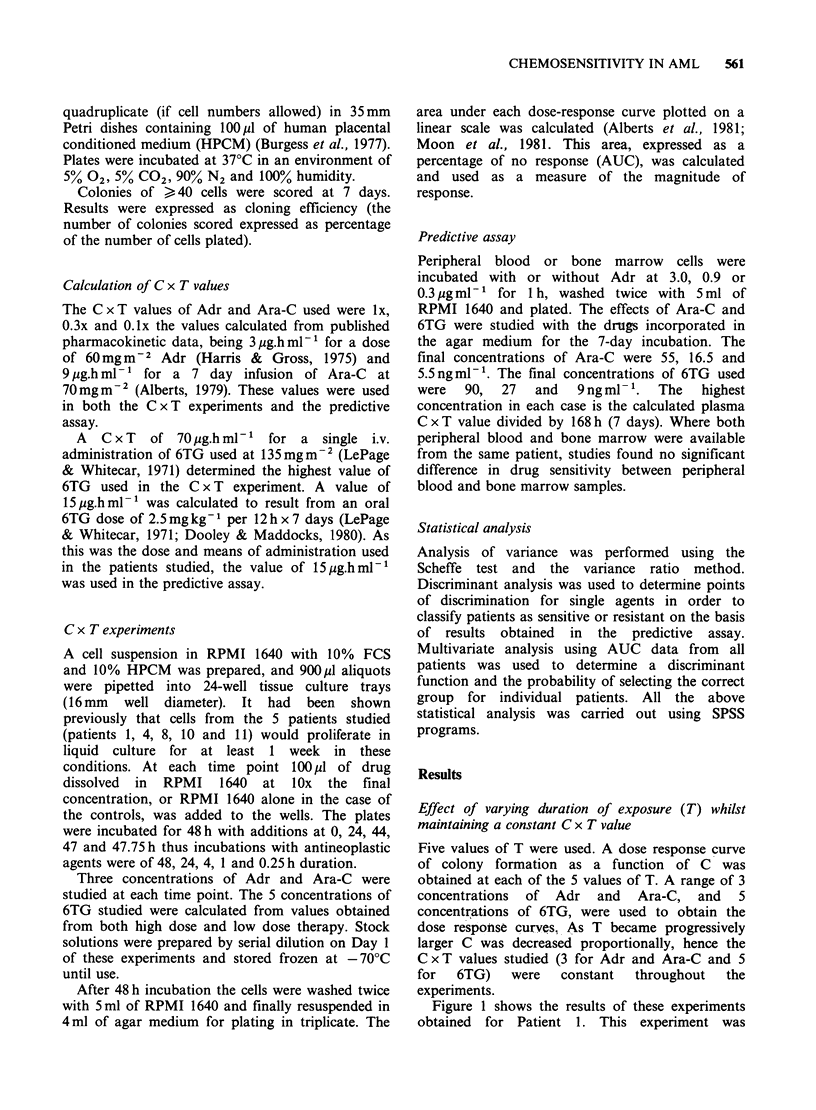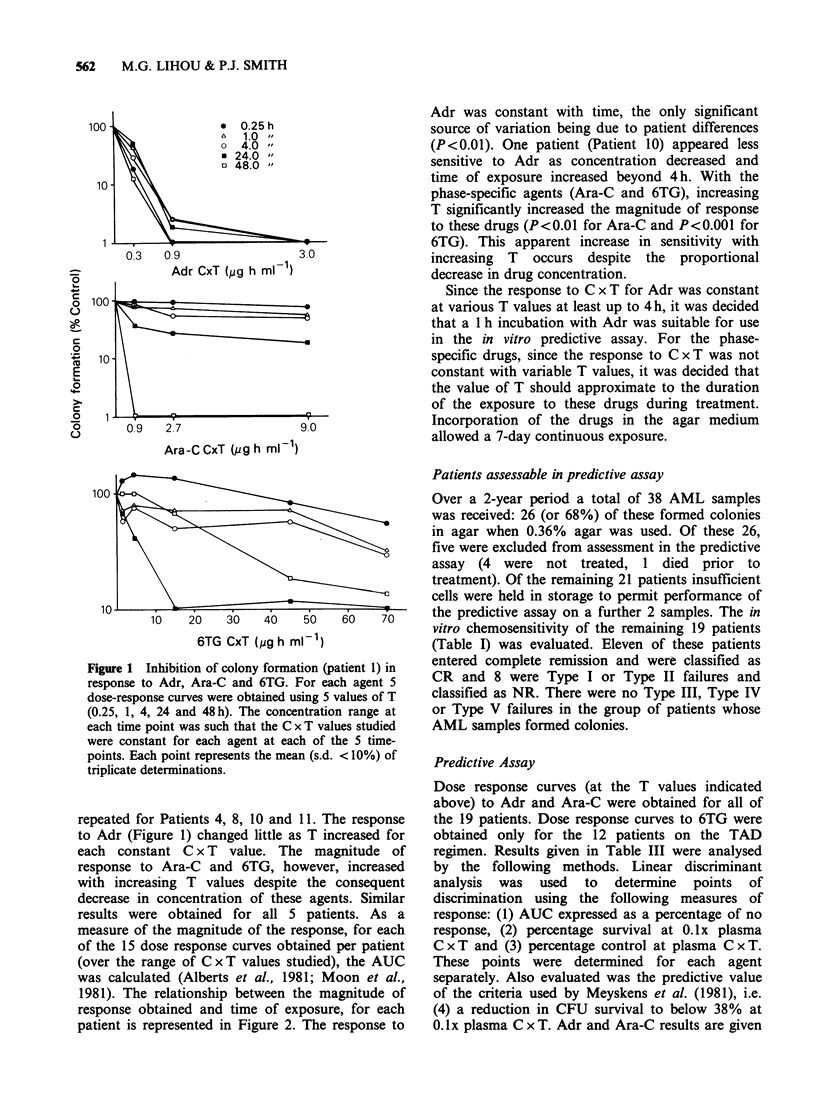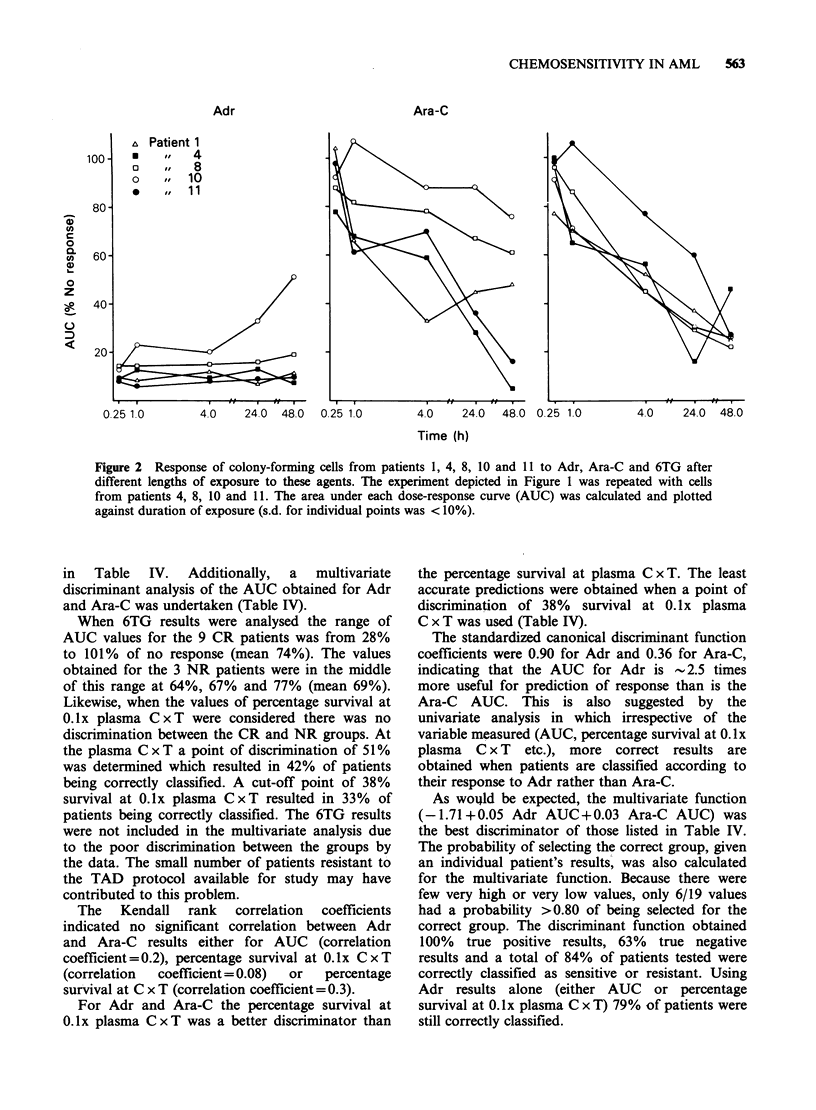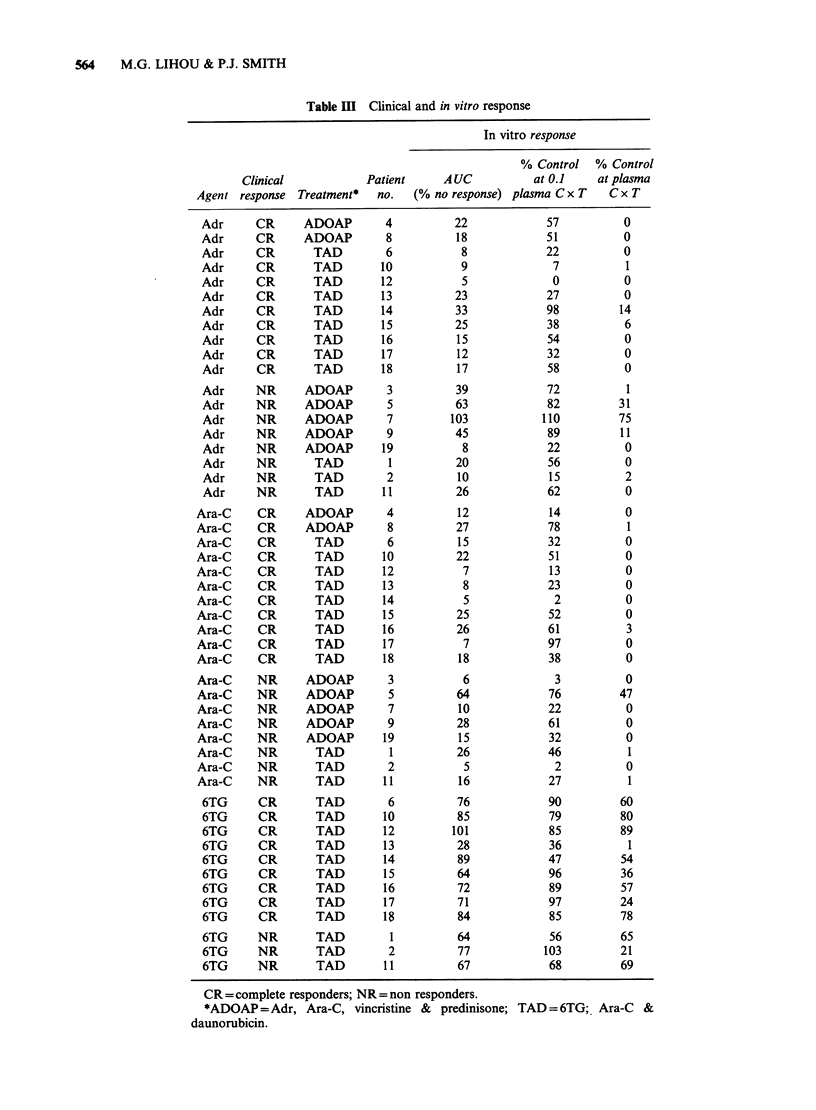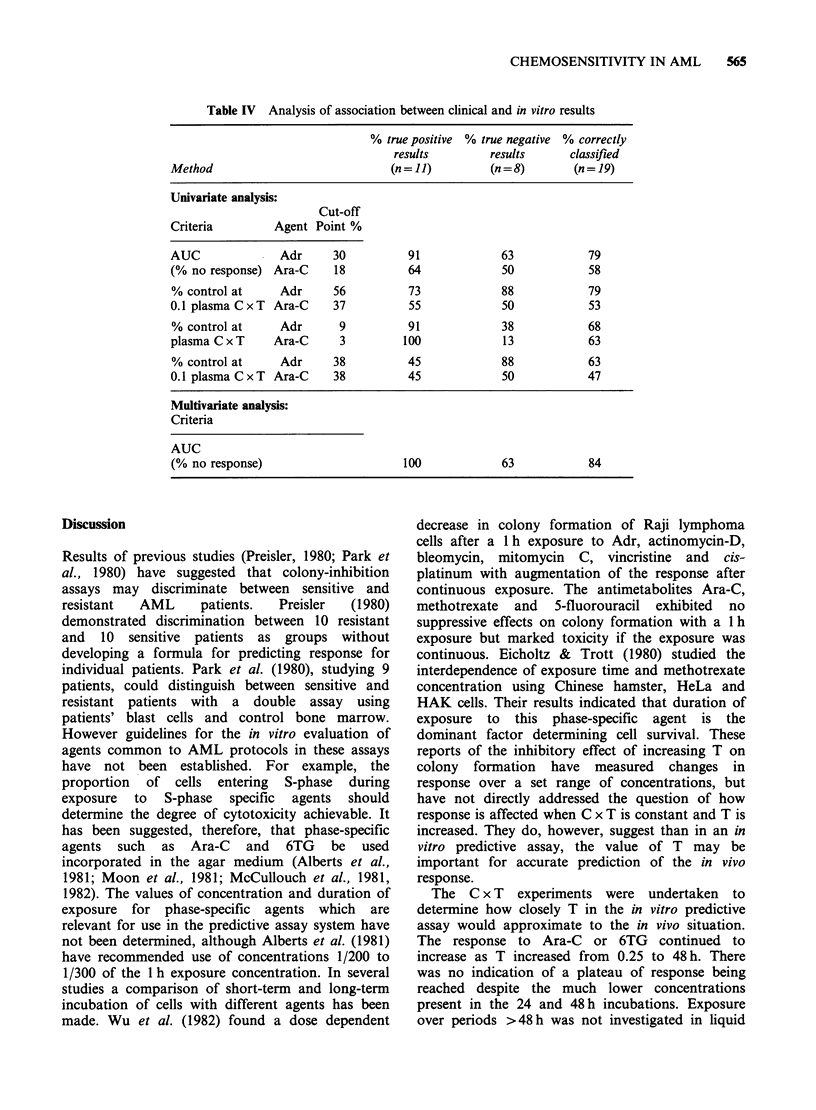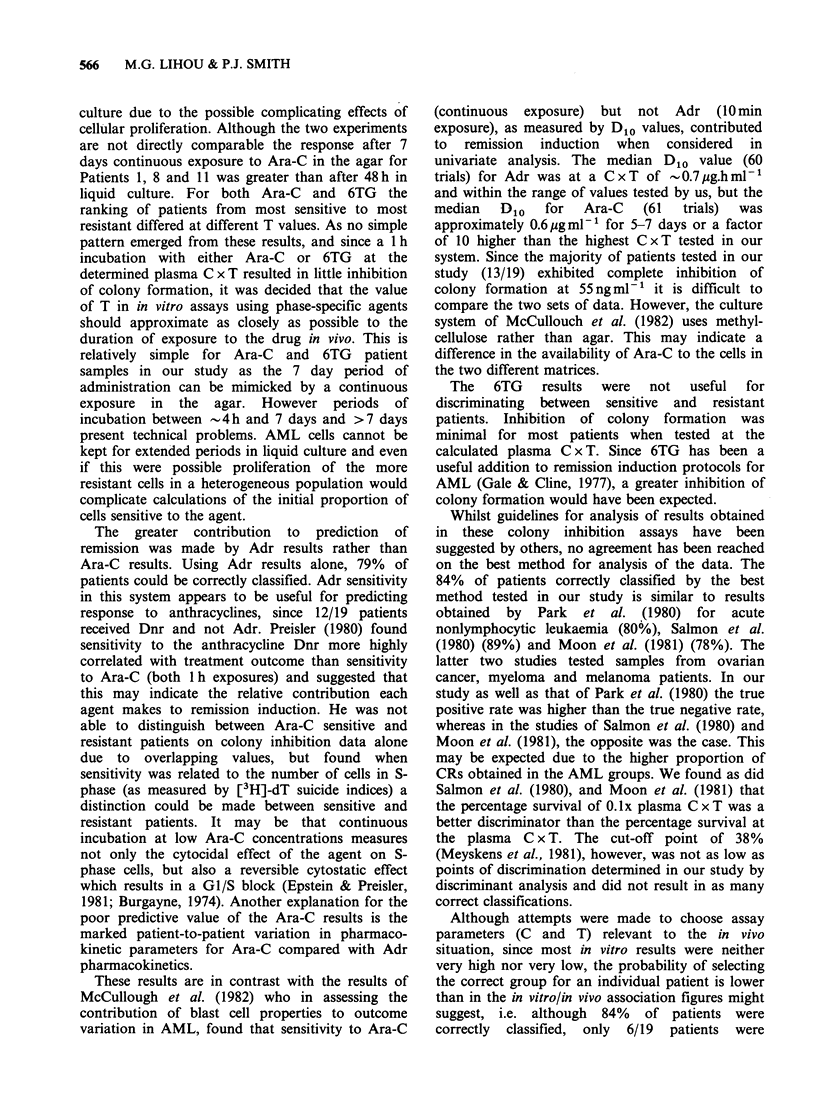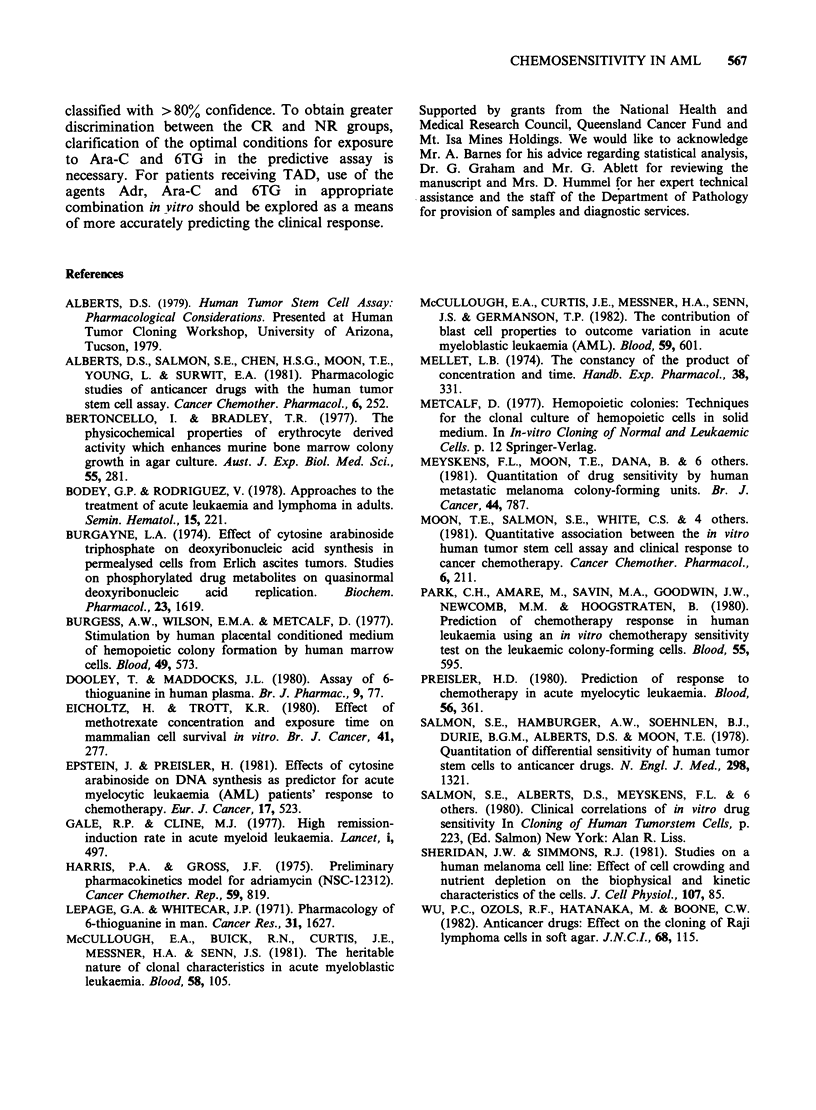# Quantitation of chemosensitivity in acute myelocytic leukaemia.

**DOI:** 10.1038/bjc.1983.229

**Published:** 1983-10

**Authors:** M. G. Lihou, P. J. Smith

## Abstract

A system for the prediction of clinical response in acute myelocytic leukaemia (AML), based on inhibition of growth of colony forming cells (CFC) was studied. If the product of initial drug concentration and time of exposure (C X T) was constant, the response to adriamycin (Adr) was constant, at T values less than 48 h. No constancy of response to the phase-specific agents cytosine arabinoside (Ara-C) and 6-thioguanine (6TG) was demonstrated with constant C X T (T value range 0.25-48 h). Hence in the predictive test, a 1 h incubation with Adr was employed whilst a continuous exposure to Ara-C and 6TG, with these drugs incorporated in the agar medium, was used. The in vitro sensitivity to Adr, Ara-C and 6TG of 19 AML patients and the predictive value of several parameters of sensitivity were evaluated. 6TG sensitivity was not useful for prediction of remission. Adr sensitivity in vitro made a greater contribution to prediction of remission than did Ara-C sensitivity. Seventy-nine percent of patients were correctly classified if Adr data alone were considered. A multivariate function including Adr and Ara-C results was obtained which resulted in 84% of patients correctly classified as sensitive or resistant to the agents received in remission-induction therapy.


*Br. J. Cancer* (1983), **48**, 559–567

Ms. 5819 October

### Quantitation of chemosensitivity in acute myelocytic leukaemia

M.G. Lihou[1] & P.J. Smith[1,2]

[1]*Queensland Institute of Medical Research and the* [2]*Royal Children's Hospital, Brisbane, 4006, Australia.*

**Summary** A system for the prediction of clinical response in acute myelocytic leukaemia (AML), based on inhibition of growth of colony forming cells (CFC) was studied. If the product of initial drug concentration and time of exposure (C×T) was constant, the response to adriamycin (Adr) was constant, at T values <48 h. No constancy of response to the phase-specific agents cytosine arabinoside (Ara-C) and 6-thioguanine (6TG) was demonstrated with constant C×T (T value range 0.25–48 h). Hence in the predictive test, a 1 h incubation with Adr was employed whilst a continuous exposure to Ara-C and 6TG, with these drugs incorporated in the agar medium, was used. The *in vitro* sensitivity to Adr, Ara-C and 6TG of 19 AML patients and the predictive value of several parameters of sensitivity were evaluated. 6TG sensitivity was not useful for prediction of remission. Adr sensitivity *in vitro* made a greater contribution to prediction of remission than did Ara-C sensitivity. Seventy-nine percent of patients were correctly classified if Adr data alone were considered. A multivariate function including Adr and Ara-C results was obtained which resulted in 84% of patients correctly classified as sensitive or resistant to the agents received in remission-induction therapy.

AML should provide a suitable model for evaluation of the use of a colony inhibition assay (Salmon *et al.*, 1978, 1980) to predict clinical response to chemotherapy, because of the ease of sample collection, the lack of requirement for enzyme digestion to obtain single cell suspensions, a high percentage of patients whose tumour cells form colonies in semi-solid media and the significant number of patients who achieve complete remission. Guidelines for determining the assay parameters of C (initial drug concentration) and T (duration of exposure), and for optimal analysis of the data obtained require resolution.

Efforts to approximate to the *in vivo* situation have led to the design of *in vitro* assays based on the pharmacokinetic parameters of the peak plasma concentration and the product of plasma concentration and time of exposure (C×T), determined in patients receiving the appropriate drugs. The *in vitro* concentration at the commencement of the incubation (C) has been derived from the plasma C×T value, the T value being constant for each agent investigated. Most investigators have used an incubation of one hour's duration (Preisler, 1980; Park *et al.*, 1980).

In many instances a constant biological response results from a constant C×T, irrespective of the individual values of C and T (Mellett, 1974). However, drugs which act specifically on S-phase cells for example, will only be capable of acting on that fraction of cells which enter S-phase during the 1 h exposure.

In this study, in order to evaluate the importance of T in the *in vitro* system, the constancy of response to a given C×T value with increasing values of T was investigated for adriamycin (Adr), cytosine arabinoside (Ara-C) and 6-thioguanine (6TG).

Having determined appropriate values of C and T for each drug, drug sensitivity profiles for a series of AML patients were obtained and then compared with the clinical response. A model was constructed for recognising sensitive and resistant patients using data obtained in these assays.

### Materials and methods

### *Patients*

Samples from 38 patients with a diagnosis of AML admitted to the Royal Brisbane Hospital or Royal Children's Hospital, Brisbane in 1981 and 1982 were studied. The clinical characteristics of those patients whose cells were studied in the predictive assay are listed in Table I. Patients received induction treatment with either Adr, Ara-C, vincristine and prednisone administered as the ADOAP regimen (Bodey & Rodriguez, 1978) or 6TG, Ara-C and daunorubicin (Dnr) administered as the TAD regimen (Gale & Cline, 1977). Complete remission was defined as disappearance of evidence of disease; normal bone marrow (blasts <5%) and normal peripheral blood smear. Patients who did not receive chemotherapy were evaluated

Correspondence: P.J. Smith

Received 21 February 1983; accepted 23 June 1983

© The Macmillan Press Ltd., 1983

**Table I** Characteristics of AML patients*

| *Patient No.* | *Sex/Age* | *Sample Tested* | *Status* |
|---|---|---|---|
| 1 | M/23 | bone marrow | Untreated |
| 2 | M/27 | bone marrow | Relapse |
| 3 | M/20 | peripheral blood | Untreated |
| 4 | F/26 | bone marrow | Untreated |
| 5 | F/18 | peripheral blood | Untreated |
| 6 | M/ 8 | peripheral blood | Untreated |
| 7 | F/27 | bone marrow | Untreated |
| 8 | F/ 9 | peripheral blood | Untreated |
| 9 | M/ 5 | peripheral blood | Untreated |
| 10 | M/41 | peripheral blood | Untreated |
| 11 | F/58 | peripheral blood | Untreated |
| 12 | M/ 2 | peripheral blood | Untreated |
| 13 | M/64 | bone marrow | Relapse |
| 14 | F/31 | peripheral blood | Untreated |
| 15* | M/28 | peripheral blood | Untreated |
| 16 | M/25 | peripheral blood | Relapse |
| 17 | M/30 | peripheral blood | Untreated |
| 18 | M/27 | bone marrow | Untreated |
| 19 | M/19 | bone marrow | Untreated |

*All patients had acute myelocytic leukaemia, with the exception of patient no. 15 (acute monocytic leukaemia).

for clonogenicity but not evaluated in the predictive assay. Patients who entered complete remission after one or two courses of treatment were classified as complete responders (CR), whilst those who did not were classified according to the criteria of Epstein & Preisler (1981). Type I and Type II failures were classified as non-responders (NR).

### *Cell preparation and storage*

Heparinised bone marrow and peripheral blood were obtained from patients with AML as part of the diagnostic procedure at presentation or relapse prior to treatment. Diluted peripheral blood (1:1 with RPMI 1640) was centrifuged on Ficoll-Paque (specific gravity, 1.077) (Pharmacia, Uppsala) at 400xg for 40 min, interface cells were collected, washed twice and resuspended in RPMI 1640 medium with 10% foetal calf serum (FCS). Bone marrow was centrifuged once at 150xg for 10 min and also resuspended in RPMI 1640 medium with 10% FCS. The fresh samples were plated in agar to determine cloning efficiency. Remaining cells were suspended in RPMI 1640 with 5% dimethylsulphoxide (DMSO) and 15% FCS, frozen and stored in liquid nitrogen. When required they were thawed rapidly to the point of phase-transition, then kept on ice, diluted slowly and washed with ice cold RPMI with 10% FCS.

### *Colony-forming assay*

The method used is a modification of standard methods for growing AML colonies (Metcalf, 1977). The agar medium consisted of 1 part bacto-agar (Difco) at 0.8%. This resulted in a final agar concentration of 0.36%. This concentration was chosen instead of the 0.3% used by Preisler (1980) and Park *et al.* (1980) since 6/22 AML samples assayed exhibited increased cloning efficiency (CE) if the agar concentration was increased (Table II).

**Table II** Effect of agar concentration on cloning efficiency (CE) of AML bone marrow of 6 individual patients

| *CE (%) (±s.d.)* | | | |
|---|---|---|---|
| *Final agar concentration (% w/v)* | | | |
| *0.27* | *0.32* | *0.36* | *0.41* |
| 0.038±0.002 | 0.10±0.02* | 0.23±0.02* | 0.29±0.03* |
| 0.60 ±0.09 | 0.58±0.16 | 2.04±0.20* | 1.96±0.01* |
| 0.06 ±0.02 | 0.05±0.04 | 3.22±0.68* | 3.10±0.22* |
| 0.00 ±0.00 | 6.80±0.44 | 8.46±0.80* | 9.22±0.62* |
| 0.00 ±0.00 | 0.00±0.00 | 15.18±0.76* | 17.18±1.51* |
| 0.00 ±0.00 | 0.17±0.02* | 0.43±0.01* | 0.29±0.02* |

*Denotes CE significantly different ($P<0.01$) from CE at 0.27% agar.

Graded agar concentrations showed that this was due to decreased dispersion of proliferating cells allowing discrete colonies to be formed. The cloning efficiency of other samples assayed exhibited no significant change with increased agar concentration. The agar was boiled for 2 min then cooled to 37°C and mixed with 1 part hypertonic medium warmed to 37°C. The hypertonic medium used was a modification of that described by Sheridan & Simmons (1981). This consisted of: FCS, 33%; Dulbecco's modified Eagle's medium (H-16, Gibco), 10 g in 215 ml water supplemented with 0.575 ml penicillin G at $2\times10^5\,U\,ml^{-1}$, and 0.375 ml streptomycin at $2\times10^5\,U\,ml^{-1}$, 29%; $NaHCO_3$, $28\,mg\,ml^{-1}$, 10%; rat erythrocyte lysate (Bertoncello & Bradley, 1981), 8.0%; HEPES buffer, $6\,mg\,ml^{-1}$ (pH 7.3), 4.0%; insulin $100\,U\,ml^{-1}$, 0.80%; L-asparaginase, $6.6\,mg\,ml^{-1}$, 0.40%; hydrocortisone, $0.18\,mg\,ml^{-1}$, 0.020%; and 14% water. The medium was sterilised by 0.45 $\mu$-membrane filtration. Cells were suspended in the agar medium at 37°C to give a final concentration of $5\times10^3$, $5\times10^4$ or $5\times10^5\,ml^{-1}$. The cell concentration for all experiments was adjusted to result in 30–100 colonies per control plate using results of preliminary platings. Aliquots (1 ml) of this suspension were plated in triplicate or quadruplicate (if cell numbers allowed) in 35 mm Petri dishes containing 100 $\mu$l of human placental conditioned medium (HPCM) (Burgess *et al.*, 1977). Plates were incubated at 37°C in an environment of 5% $O_2$, 5% $CO_2$, 90% $N_2$ and 100% humidity.

Colonies of $\geqslant$40 cells were scored at 7 days. Results were expressed as cloning efficiency (the number of colonies scored expressed as percentage of the number of cells plated).

*Calculation of C × T values*

The C × T values of Adr and Ara-C used were 1x, 0.3x and 0.1x the values calculated from published pharmacokinetic data, being 3 $\mu$g.h ml$^{-1}$ for a dose of 60 mg m$^{-2}$ Adr (Harris & Gross, 1975) and 9 $\mu$g.h ml$^{-1}$ for a 7 day infusion of Ara-C at 70 mg m$^{-2}$ (Alberts, 1979). These values were used in both the C × T experiments and the predictive assay.

A C × T of 70 $\mu$g.h ml$^{-1}$ for a single i.v. administration of 6TG used at 135 mg m$^{-2}$ (LePage & Whitecar, 1971) determined the highest value of 6TG used in the C × T experiment. A value of 15 $\mu$g.h ml$^{-1}$ was calculated to result from an oral 6TG dose of 2.5 mg kg$^{-1}$ per 12 h × 7 days (LePage & Whitecar, 1971; Dooley & Maddocks, 1980). As this was the dose and means of administration used in the patients studied, the value of 15 $\mu$g.h ml$^{-1}$ was used in the predictive assay.

*C × T experiments*

A cell suspension in RPMI 1640 with 10% FCS and 10% HPCM was prepared, and 900 $\mu$l aliquots were pipetted into 24-well tissue culture trays (16 mm well diameter). It had been shown previously that cells from the 5 patients studied (patients 1, 4, 8, 10 and 11) would proliferate in liquid culture for at least 1 week in these conditions. At each time point 100 $\mu$l of drug dissolved in RPMI 1640 at 10x the final concentration, or RPMI 1640 alone in the case of the controls, was added to the wells. The plates were incubated for 48 h with additions at 0, 24, 44, 47 and 47.75 h thus incubations with antineoplastic agents were of 48, 24, 4, 1 and 0.25 h duration.

Three concentrations of Adr and Ara-C were studied at each time point. The 5 concentrations of 6TG studied were calculated from values obtained from both high dose and low dose therapy. Stock solutions were prepared by serial dilution on Day 1 of these experiments and stored frozen at −70°C until use.

After 48 h incubation the cells were washed twice with 5 ml of RPMI 1640 and finally resuspended in 4 ml of agar medium for plating in triplicate. The area under each dose-response curve plotted on a linear scale was calculated (Alberts *et al.*, 1981; Moon *et al.*, 1981. This area, expressed as a percentage of no response (AUC), was calculated and used as a measure of the magnitude of response.

*Predictive assay*

Peripheral blood or bone marrow cells were incubated with or without Adr at 3.0, 0.9 or 0.3 $\mu$g ml$^{-1}$ for 1 h, washed twice with 5 ml of RPMI 1640 and plated. The effects of Ara-C and 6TG were studied with the drugs incorporated in the agar medium for the 7-day incubation. The final concentrations of Ara-C were 55, 16.5 and 5.5 ng ml$^{-1}$. The final concentrations of 6TG used were 90, 27 and 9 ng ml$^{-1}$. The highest concentration in each case is the calculated plasma C × T value divided by 168 h (7 days). Where both peripheral blood and bone marrow were available from the same patient, studies found no significant difference in drug sensitivity between peripheral blood and bone marrow samples.

*Statistical analysis*

Analysis of variance was performed using the Scheffe test and the variance ratio method. Discriminant analysis was used to determine points of discrimination for single agents in order to classify patients as sensitive or resistant on the basis of results obtained in the predictive assay. Multivariate analysis using AUC data from all patients was used to determine a discriminant function and the probability of selecting the correct group for individual patients. All the above statistical analysis was carried out using SPSS programs.

### Results

*Effect of varying duration of exposure (T) whilst maintaining a constant C × T value*

Five values of T were used. A dose response curve of colony formation as a function of C was obtained at each of the 5 values of T. A range of 3 concentrations of Adr and Ara-C, and 5 concentrations of 6TG, were used to obtain the dose response curves. As T became progressively larger C was decreased proportionally, hence the C × T values studied (3 for Adr and Ara-C and 5 for 6TG) were constant throughout the experiments.

Figure 1 shows the results of these experiments obtained for Patient 1. This experiment was

**Figure 1** Inhibition of colony formation (patient 1) in response to Adr, Ara-C and 6TG. For each agent 5 dose-response curves were obtained using 5 values of T (0.25, 1, 4, 24 and 48 h). The concentration range at each time point was such that the C x T values studied were constant for each agent at each of the 5 time-points. Each point represents the mean (s.d. <10%) of triplicate determinations.

repeated for Patients 4, 8, 10 and 11. The response to Adr (Figure 1) changed little as T increased for each constant C x T value. The magnitude of response to Ara-C and 6TG, however, increased with increasing T values despite the consequent decrease in concentration of these agents. Similar results were obtained for all 5 patients. As a measure of the magnitude of the response, for each of the 15 dose response curves obtained per patient (over the range of C x T values studied), the AUC was calculated (Alberts *et al.*, 1981; Moon *et al.*, 1981). The relationship between the magnitude of response obtained and time of exposure, for each patient is represented in Figure 2. The response to Adr was constant with time, the only significant source of variation being due to patient differences ($P<0.01$). One patient (Patient 10) appeared less sensitive to Adr as concentration decreased and time of exposure increased beyond 4 h. With the phase-specific agents (Ara-C and 6TG), increasing T significantly increased the magnitude of response to these drugs ($P<0.01$ for Ara-C and $P<0.001$ for 6TG). This apparent increase in sensitivity with increasing T occurs despite the proportional decrease in drug concentration.

Since the response to C x T for Adr was constant at various T values at least up to 4 h, it was decided that a 1 h incubation with Adr was suitable for use in the *in vitro* predictive assay. For the phase-specific drugs, since the response to C x T was not constant with variable T values, it was decided that the value of T should approximate to the duration of the exposure to these drugs during treatment. Incorporation of the drugs in the agar medium allowed a 7-day continuous exposure.

*Patients assessable in predictive assay*

Over a 2-year period a total of 38 AML samples was received: 26 (or 68%) of these formed colonies in agar when 0.36% agar was used. Of these 26, five were excluded from assessment in the predictive assay (4 were not treated, 1 died prior to treatment). Of the remaining 21 patients insufficient cells were held in storage to permit performance of the predictive assay on a further 2 samples. The *in vitro* chemosensitivity of the remaining 19 patients (Table I) was evaluated. Eleven of these patients entered complete remission and were classified as CR and 8 were Type I or Type II failures and classified as NR. There were no Type III, Type IV or Type V failures in the group of patients whose AML samples formed colonies.

*Predictive Assay*

Dose response curves (at the T values indicated above) to Adr and Ara-C were obtained for all of the 19 patients. Dose response curves to 6TG were obtained only for the 12 patients on the TAD regimen. Results given in Table III were analysed by the following methods. Linear discriminant analysis was used to determine points of discrimination using the following measures of response: (1) AUC expressed as a percentage of no response, (2) percentage survival at 0.1x plasma C x T and (3) percentage control at plasma C x T. These points were determined for each agent separately. Also evaluated was the predictive value of the criteria used by Meyskens *et al.* (1981), i.e. (4) a reduction in CFU survival to below 38% at 0.1x plasma C x T. Adr and Ara-C results are given

**Figure 2** Response of colony-forming cells from patients 1, 4, 8, 10 and 11 to Adr, Ara-C and 6TG after different lengths of exposure to these agents. The experiment depicted in Figure 1 was repeated with cells from patients 4, 8, 10 and 11. The area under each dose-response curve (AUC) was calculated and plotted against duration of exposure (s.d. for individual points was <10%).

in Table IV. Additionally, a multivariate discriminant analysis of the AUC obtained for Adr and Ara-C was undertaken (Table IV).

When 6TG results were analysed the range of AUC values for the 9 CR patients was from 28% to 101% of no response (mean 74%). The values obtained for the 3 NR patients were in the middle of this range at 64%, 67% and 77% (mean 69%). Likewise, when the values of percentage survival at 0.1x plasma C x T were considered there was no discrimination between the CR and NR groups. At the plasma C x T a point of discrimination of 51% was determined which resulted in 42% of patients being correctly classified. A cut-off point of 38% survival at 0.1x plasma C x T resulted in 33% of patients being correctly classified. The 6TG results were not included in the multivariate analysis due to the poor discrimination between the groups by the data. The small number of patients resistant to the TAD protocol available for study may have contributed to this problem.

The Kendall rank correlation coefficients indicated no significant correlation between Adr and Ara-C results either for AUC (correlation coefficient = 0.2), percentage survival at 0.1x C x T (correlation coefficient = 0.08) or percentage survival at C x T (correlation coefficient = 0.3).

For Adr and Ara-C the percentage survival at 0.1x plasma C x T was a better discriminator than the percentage survival at plasma C x T. The least accurate predictions were obtained when a point of discrimination of 38% survival at 0.1x plasma C x T was used (Table IV).

The standardized canonical discriminant function coefficients were 0.90 for Adr and 0.36 for Ara-C, indicating that the AUC for Adr is ~2.5 times more useful for prediction of response than is the Ara-C AUC. This is also suggested by the univariate analysis in which irrespective of the variable measured (AUC, percentage survival at 0.1x plasma C x T etc.), more correct results are obtained when patients are classified according to their response to Adr rather than Ara-C.

As would be expected, the multivariate function (−1.71 + 0.05 Adr AUC + 0.03 Ara-C AUC) was the best discriminator of those listed in Table IV. The probability of selecting the correct group, given an individual patient's results, was also calculated for the multivariate function. Because there were few very high or very low values, only 6/19 values had a probability >0.80 of being selected for the correct group. The discriminant function obtained 100% true positive results, 63% true negative results and a total of 84% of patients tested were correctly classified as sensitive or resistant. Using Adr results alone (either AUC or percentage survival at 0.1x plasma C x T) 79% of patients were still correctly classified.

**Table III** Clinical and *in vitro* response

| Agent | Clinical response | Treatment* | Patient no. | In vitro *response* AUC (% no response) | % Control at 0.1 plasma C × T | % Control at plasma C × T |
|---|---|---|---|---|---|---|
| Adr | CR | ADOAP | 4 | 22 | 57 | 0 |
| Adr | CR | ADOAP | 8 | 18 | 51 | 0 |
| Adr | CR | TAD | 6 | 8 | 22 | 0 |
| Adr | CR | TAD | 10 | 9 | 7 | 1 |
| Adr | CR | TAD | 12 | 5 | 0 | 0 |
| Adr | CR | TAD | 13 | 23 | 27 | 0 |
| Adr | CR | TAD | 14 | 33 | 98 | 14 |
| Adr | CR | TAD | 15 | 25 | 38 | 6 |
| Adr | CR | TAD | 16 | 15 | 54 | 0 |
| Adr | CR | TAD | 17 | 12 | 32 | 0 |
| Adr | CR | TAD | 18 | 17 | 58 | 0 |
| Adr | NR | ADOAP | 3 | 39 | 72 | 1 |
| Adr | NR | ADOAP | 5 | 63 | 82 | 31 |
| Adr | NR | ADOAP | 7 | 103 | 110 | 75 |
| Adr | NR | ADOAP | 9 | 45 | 89 | 11 |
| Adr | NR | ADOAP | 19 | 8 | 22 | 0 |
| Adr | NR | TAD | 1 | 20 | 56 | 0 |
| Adr | NR | TAD | 2 | 10 | 15 | 2 |
| Adr | NR | TAD | 11 | 26 | 62 | 0 |
| Ara-C | CR | ADOAP | 4 | 12 | 14 | 0 |
| Ara-C | CR | ADOAP | 8 | 27 | 78 | 1 |
| Ara-C | CR | TAD | 6 | 15 | 32 | 0 |
| Ara-C | CR | TAD | 10 | 22 | 51 | 0 |
| Ara-C | CR | TAD | 12 | 7 | 13 | 0 |
| Ara-C | CR | TAD | 13 | 8 | 23 | 0 |
| Ara-C | CR | TAD | 14 | 5 | 2 | 0 |
| Ara-C | CR | TAD | 15 | 25 | 52 | 0 |
| Ara-C | CR | TAD | 16 | 26 | 61 | 3 |
| Ara-C | CR | TAD | 17 | 7 | 97 | 0 |
| Ara-C | CR | TAD | 18 | 18 | 38 | 0 |
| Ara-C | NR | ADOAP | 3 | 6 | 3 | 0 |
| Ara-C | NR | ADOAP | 5 | 64 | 76 | 47 |
| Ara-C | NR | ADOAP | 7 | 10 | 22 | 0 |
| Ara-C | NR | ADOAP | 9 | 28 | 61 | 0 |
| Ara-C | NR | ADOAP | 19 | 15 | 32 | 0 |
| Ara-C | NR | TAD | 1 | 26 | 46 | 1 |
| Ara-C | NR | TAD | 2 | 5 | 2 | 0 |
| Ara-C | NR | TAD | 11 | 16 | 27 | 1 |
| 6TG | CR | TAD | 6 | 76 | 90 | 60 |
| 6TG | CR | TAD | 10 | 85 | 79 | 80 |
| 6TG | CR | TAD | 12 | 101 | 85 | 89 |
| 6TG | CR | TAD | 13 | 28 | 36 | 1 |
| 6TG | CR | TAD | 14 | 89 | 47 | 54 |
| 6TG | CR | TAD | 15 | 64 | 96 | 36 |
| 6TG | CR | TAD | 16 | 72 | 89 | 57 |
| 6TG | CR | TAD | 17 | 71 | 97 | 24 |
| 6TG | CR | TAD | 18 | 84 | 85 | 78 |
| 6TG | NR | TAD | 1 | 64 | 56 | 65 |
| 6TG | NR | TAD | 2 | 77 | 103 | 21 |
| 6TG | NR | TAD | 11 | 67 | 68 | 69 |

CR = complete responders; NR = non responders.
*ADOAP = Adr, Ara-C, vincristine & predinisone; TAD = 6TG; Ara-C & daunorubicin.

**Table IV** Analysis of association between clinical and *in vitro* results

| *Method* | | | *% true positive results (n=11)* | *% true negative results (n=8)* | *% correctly classified (n=19)* |
|---|---|---|---|---|---|
| **Univariate analysis:** | | | | | |
| Criteria | Agent | Cut-off Point % | | | |
| AUC | Adr | 30 | 91 | 63 | 79 |
| (% no response) | Ara-C | 18 | 64 | 50 | 58 |
| % control at | Adr | 56 | 73 | 88 | 79 |
| 0.1 plasma C x T | Ara-C | 37 | 55 | 50 | 53 |
| % control at | Adr | 9 | 91 | 38 | 68 |
| plasma C x T | Ara-C | 3 | 100 | 13 | 63 |
| % control at | Adr | 38 | 45 | 88 | 63 |
| 0.1 plasma C x T | Ara-C | 38 | 45 | 50 | 47 |
| **Multivariate analysis:** | | | | | |
| Criteria | | | | | |
| AUC (% no response) | | | 100 | 63 | 84 |

### Discussion

Results of previous studies (Preisler, 1980; Park *et al.*, 1980) have suggested that colony-inhibition assays may discriminate between sensitive and resistant AML patients. Preisler (1980) demonstrated discrimination between 10 resistant and 10 sensitive patients as groups without developing a formula for predicting response for individual patients. Park *et al.* (1980), studying 9 patients, could distinguish between sensitive and resistant patients with a double assay using patients' blast cells and control bone marrow. However guidelines for the *in vitro* evaluation of agents common to AML protocols in these assays have not been established. For example, the proportion of cells entering S-phase during exposure to S-phase specific agents should determine the degree of cytotoxicity achievable. It has been suggested, therefore, that phase-specific agents such as Ara-C and 6TG be used incorporated in the agar medium (Alberts *et al.*, 1981; Moon *et al.*, 1981; McCullouch *et al.*, 1981, 1982). The values of concentration and duration of exposure for phase-specific agents which are relevant for use in the predictive assay system have not been determined, although Alberts *et al.* (1981) have recommended use of concentrations 1/200 to 1/300 of the 1 h exposure concentration. In several studies a comparison of short-term and long-term incubation of cells with different agents has been made. Wu *et al.* (1982) found a dose dependent decrease in colony formation of Raji lymphoma cells after a 1 h exposure to Adr, actinomycin-D, bleomycin, mitomycin C, vincristine and *cis*-platinum with augmentation of the response after continuous exposure. The antimetabolites Ara-C, methotrexate and 5-fluorouracil exhibited no suppressive effects on colony formation with a 1 h exposure but marked toxicity if the exposure was continuous. Eicholtz & Trott (1980) studied the interdependence of exposure time and methotrexate concentration using Chinese hamster, HeLa and HAK cells. Their results indicated that duration of exposure to this phase-specific agent is the dominant factor determining cell survival. These reports of the inhibitory effect of increasing T on colony formation have measured changes in response over a set range of concentrations, but have not directly addressed the question of how response is affected when C x T is constant and T is increased. They do, however, suggest than in an *in vitro* predictive assay, the value of T may be important for accurate prediction of the *in vivo* response.

The C x T experiments were undertaken to determine how closely T in the *in vitro* predictive assay would approximate to the *in vivo* situation. The response to Ara-C or 6TG continued to increase as T increased from 0.25 to 48 h. There was no indication of a plateau of response being reached despite the much lower concentrations present in the 24 and 48 h incubations. Exposure over periods >48 h was not investigated in liquid culture due to the possible complicating effects of cellular proliferation. Although the two experiments are not directly comparable the response after 7 days continuous exposure to Ara-C in the agar for Patients 1, 8 and 11 was greater than after 48 h in liquid culture. For both Ara-C and 6TG the ranking of patients from most sensitive to most resistant differed at different T values. As no simple pattern emerged from these results, and since a 1 h incubation with either Ara-C or 6TG at the determined plasma C x T resulted in little inhibition of colony formation, it was decided that the value of T in *in vitro* assays using phase-specific agents should approximate as closely as possible to the duration of exposure to the drug *in vivo*. This is relatively simple for Ara-C and 6TG patient samples in our study as the 7 day period of administration can be mimicked by a continuous exposure in the agar. However periods of incubation between ~4 h and 7 days and >7 days present technical problems. AML cells cannot be kept for extended periods in liquid culture and even if this were possible proliferation of the more resistant cells in a heterogeneous population would complicate calculations of the initial proportion of cells sensitive to the agent.

The greater contribution to prediction of remission was made by Adr results rather than Ara-C results. Using Adr results alone, 79% of patients could be correctly classified. Adr sensitivity in this system appears to be useful for predicting response to anthracyclines, since 12/19 patients received Dnr and not Adr. Preisler (1980) found sensitivity to the anthracycline Dnr more highly correlated with treatment outcome than sensitivity to Ara-C (both 1 h exposures) and suggested that this may indicate the relative contribution each agent makes to remission induction. He was not able to distinguish between Ara-C sensitive and resistant patients on colony inhibition data alone due to overlapping values, but found when sensitivity was related to the number of cells in S-phase (as measured by [$^3$H]-dT suicide indices) a distinction could be made between sensitive and resistant patients. It may be that continuous incubation at low Ara-C concentrations measures not only the cytocidal effect of the agent on S-phase cells, but also a reversible cytostatic effect which results in a G1/S block (Epstein & Preisler, 1981; Burgayne, 1974). Another explanation for the poor predictive value of the Ara-C results is the marked patient-to-patient variation in pharmacokinetic parameters for Ara-C compared with Adr pharmacokinetics.

These results are in contrast with the results of McCullough *et al.* (1982) who in assessing the contribution of blast cell properties to outcome variation in AML, found that sensitivity to Ara-C (continuous exposure) but not Adr (10 min exposure), as measured by $D_{10}$ values, contributed to remission induction when considered in univariate analysis. The median $D_{10}$ value (60 trials) for Adr was at a C x T of ~0.7 $\mu$g.h ml$^{-1}$ and within the range of values tested by us, but the median $D_{10}$ for Ara-C (61 trials) was approximately 0.6 $\mu$g ml$^{-1}$ for 5–7 days or a factor of 10 higher than the highest C x T tested in our system. Since the majority of patients tested in our study (13/19) exhibited complete inhibition of colony formation at 55 ng ml$^{-1}$ it is difficult to compare the two sets of data. However, the culture system of McCullouch *et al.* (1982) uses methylcellulose rather than agar. This may indicate a difference in the availability of Ara-C to the cells in the two different matrices.

The 6TG results were not useful for discriminating between sensitive and resistant patients. Inhibition of colony formation was minimal for most patients when tested at the calculated plasma C x T. Since 6TG has been a useful addition to remission induction protocols for AML (Gale & Cline, 1977), a greater inhibition of colony formation would have been expected.

Whilst guidelines for analysis of results obtained in these colony inhibition assays have been suggested by others, no agreement has been reached on the best method for analysis of the data. The 84% of patients correctly classified by the best method tested in our study is similar to results obtained by Park *et al.* (1980) for acute nonlymphocytic leukaemia (80%), Salmon *et al.* (1980) (89%) and Moon *et al.* (1981) (78%). The latter two studies tested samples from ovarian cancer, myeloma and melanoma patients. In our study as well as that of Park *et al.* (1980) the true positive rate was higher than the true negative rate, whereas in the studies of Salmon *et al.* (1980) and Moon *et al.* (1981), the opposite was the case. This may be expected due to the higher proportion of CRs obtained in the AML groups. We found as did Salmon *et al.* (1980), and Moon *et al.* (1981) that the percentage survival of 0.1x plasma C x T was a better discriminator than the percentage survival at the plasma C x T. The cut-off point of 38% (Meyskens *et al.*, 1981), however, was not as low as points of discrimination determined in our study by discriminant analysis and did not result in as many correct classifications.

Although attempts were made to choose assay parameters (C and T) relevant to the *in vivo* situation, since most *in vitro* results were neither very high nor very low, the probability of selecting the correct group for an individual patient is lower than in the *in vitro*/*in vivo* association figures might suggest, i.e. although 84% of patients were correctly classified, only 6/19 patients were classified with >80% confidence. To obtain greater discrimination between the CR and NR groups, clarification of the optimal conditions for exposure to Ara-C and 6TG in the predictive assay is necessary. For patients receiving TAD, use of the agents Adr, Ara-C and 6TG in appropriate combination *in vitro* should be explored as a means of more accurately predicting the clinical response.

Supported by grants from the National Health and Medical Research Council, Queensland Cancer Fund and Mt. Isa Mines Holdings. We would like to acknowledge Mr. A. Barnes for his advice regarding statistical analysis, Dr. G. Graham and Mr. G. Ablett for reviewing the manuscript and Mrs. D. Hummel for her expert technical assistance and the staff of the Department of Pathology for provision of samples and diagnostic services.

### References

ALBERTS, D.S. (1979). *Human Tumor Stem Cell Assay: Pharmacological Considerations*. Presented at Human Tumor Cloning Workshop, University of Arizona, Tucson, 1979.

ALBERTS, D.S., SALMON, S.E., CHEN, H.S.G., MOON, T.E., YOUNG, L. & SURWIT, E.A. (1981). Pharmacologic studies of anticancer drugs with the human tumor stem cell assay. *Cancer Chemother. Pharmacol.*, **6**, 252.

BERTONCELLO, I. & BRADLEY, T.R. (1977). The physicochemical properties of erythrocyte derived activity which enhances murine bone marrow colony growth in agar culture. *Aust. J. Exp. Biol. Med. Sci.*, **55**, 281.

BODEY, G.P. & RODRIGUEZ, V. (1978). Approaches to the treatment of acute leukaemia and lymphoma in adults. *Semin. Hematol.*, **15**, 221.

BURGAYNE, L.A. (1974). Effect of cytosine arabinoside triphosphate on deoxyribonucleic acid synthesis in permealysed cells from Erlich ascites tumors. Studies on phosphorylated drug metabolites on quasinormal deoxyribonucleic acid replication. *Biochem. Pharmacol.*, **23**, 1619.

BURGESS, A.W., WILSON, E.M.A. & METCALF, D. (1977). Stimulation by human placental conditioned medium of hemopoietic colony formation by human marrow cells. *Blood*, **49**, 573.

DOOLEY, T. & MADDOCKS, J.L. (1980). Assay of 6-thioguanine in human plasma. *Br. J. Pharmac.*, **9**, 77.

EICHOLTZ, H. & TROTT, K.R. (1980). Effect of methotrexate concentration and exposure time on mammalian cell survival *in vitro*. *Br. J. Cancer*, **41**, 277.

EPSTEIN, J. & PREISLER, H. (1981). Effects of cytosine arabinoside on DNA synthesis as predictor for acute myelocytic leukaemia (AML) patients' response to chemotherapy. *Eur. J. Cancer*, **17**, 523.

GALE, R.P. & CLINE, M.J. (1977). High remission-induction rate in acute myeloid leukaemia. *Lancet*, **i**, 497.

HARRIS, P.A. & GROSS, J.F. (1975). Preliminary pharmacokinetics model for adriamycin (NSC-12312). *Cancer Chemother. Rep.*, **59**, 819.

LEPAGE, G.A. & WHITECAR, J.P. (1971). Pharmacology of 6-thioguanine in man. *Cancer Res.*, **31**, 1627.

McCULLOUGH, E.A., BUICK, R.N., CURTIS, J.E., MESSNER, H.A. & SENN, J.S. (1981). The heritable nature of clonal characteristics in acute myeloblastic leukaemia. *Blood*, **58**, 105.

McCULLOUGH, E.A., CURTIS, J.E., MESSNER, H.A., SENN, J.S. & GERMANSON, T.P. (1982). The contribution of blast cell properties to outcome variation in acute myeloblastic leukaemia (AML). *Blood*, **59**, 601.

MELLET, L.B. (1974). The constancy of the product of concentration and time. *Handb. Exp. Pharmacol.*, **38**, 331.

METCALF, D. (1977). Hemopoietic colonies: Techniques for the clonal culture of hemopoietic cells in solid medium. In *In-vitro Cloning of Normal and Leukaemic Cells*. p. 12 Springer-Verlag.

MEYSKENS, F.L., MOON, T.E., DANA, B. & 6 others. (1981). Quantitation of drug sensitivity by human metastatic melanoma colony-forming units. *Br. J. Cancer*, **44**, 787.

MOON, T.E., SALMON, S.E., WHITE, C.S. & 4 others. (1981). Quantitative association between the *in vitro* human tumor stem cell assay and clinical response to cancer chemotherapy. *Cancer Chemother. Pharmacol.*, **6**, 211.

PARK, C.H., AMARE, M., SAVIN, M.A., GOODWIN, J.W., NEWCOMB, M.M. & HOOGSTRATEN, B. (1980). Prediction of chemotherapy response in human leukaemia using an *in vitro* chemotherapy sensitivity test on the leukaemic colony-forming cells. *Blood*, **55**, 595.

PREISLER, H.D. (1980). Prediction of response to chemotherapy in acute myelocytic leukaemia. *Blood*, **56**, 361.

SALMON, S.E., HAMBURGER, A.W., SOEHNLEN, B.J., DURIE, B.G.M., ALBERTS, D.S. & MOON, T.E. (1978). Quantitation of differential sensitivity of human tumor stem cells to anticancer drugs. *N. Engl. J. Med.*, **298**, 1321.

SALMON, S.E., ALBERTS, D.S., MEYSKENS, F.L. & 6 others. (1980). Clinical correlations of *in vitro* drug sensitivity In *Cloning of Human Tumorstem Cells*, p. 223, (Ed. Salmon) New York: Alan R. Liss.

SHERIDAN, J.W. & SIMMONS, R.J. (1981). Studies on a human melanoma cell line: Effect of cell crowding and nutrient depletion on the biophysical and kinetic characteristics of the cells. *J. Cell Physiol.*, **107**, 85.

WU, P.C., OZOLS, R.F., HATANAKA, M. & BOONE, C.W. (1982). Anticancer drugs: Effect on the cloning of Raji lymphoma cells in soft agar. *J.N.C.I.*, **68**, 115.